# RT-based combination therapy for brain metastasis from NSCLC with non-EGFR mutation/ALK gene rearrangement: A network meta-analysis

**DOI:** 10.3389/fonc.2022.1024833

**Published:** 2022-11-28

**Authors:** Min Wu, Jun Jiang, Xuewen Zhang, Jie Chen, Qiaomei Chang, Rong Chen

**Affiliations:** ^1^ Department of Oncology, Graduate School of Qinghai University, Qinghai, China; ^2^ Division III, Department of Medical Oncology, Affiliated Hospital of Qinghai University, Qinghai, China; ^3^ Department of Radiotherapy, Affiliated Hospital of Qinghai University, Qinghai, China

**Keywords:** radiotherapy, brain metastasis, NSCLC, bayesian network meta-analysis, neuro-oncology

## Abstract

**Introduction:**

Radiotherapy (RT) is currently the main treatment for brain metastases (BMs) from non-small cell lung cancer (NSCLC). Due to the short survival time and obvious adverse reactions of RT, we urgently need more appropriate treatment. This network meta-analysis reviewed the efficacy and adverse effects of radiotherapy-based combination therapy for patients without targeted epidermal growth factor receptor (EGFR) mutations/anaplastic lymphoma kinase (ALK) gene rearrangement NSCLC BMs, to screen out the therapy with the best efficacy.

**Methods:**

PubMed, Embase, Web of Science, and Cochrane Library were searched from the earliest publication date available to 1 April 2022. STATA15.0 was used to conduct heterogeneity analysis, sensitivity analysis, forest plot analysis, and publication bias analysis.

**Results:**

A total of 28 studies, involving 3707 patients were included in the Bayesian network meta-analysis. In the limited paired meta-analysis for head-to-head comparative trials, compared with RT-based combination therapy, RT combined with Immune checkpoint inhibitors (ICIs) showed significant overall survival (OS) benefit (HR 0.65, 95%CI 0.47–0.9, p<0.01), RT combined with ICIs showed a non-significant difference for intracranial progression-free survival (iPFS) (HR 0.76, 95%CI 0.27–2.27, p<0.01) and progression-free survival (PFS) (HR 0.9, 95%CI 0.36–2.37, p<0.01). In addition, according to the ranking results, compared with RT combined with chemotherapy(CT) or with targeted therapy(TT), RT combined with ICIs might be the best treatment mode for OS(ICIs+RT vs CT+RT vs TT+RT; 91.9% vs. 27.8% vs. 29.3%, iPFS (ICIs+RT vs CT+RT vs TT+RT, 46.9% vs 25.2% vs 25.6%) and PFS (ICIs+RT vs CT+RT vs TT+RT, 36.2% vs 31% vs 36.5%).

**Conclusions:**

RT combined with ICIs might be the best treatment mode to prolong the OS for BMs from NSCLC with non-EGFR mutation/ALK gene rearrangement.

**Review Registration:**

https://www.crd.york.ac.uk/prospero/display_record.php?ID=CRD42022350065, identifier (CRD42022350065)

## Introduction

Brain metastases (BMs) are a common complication of non-small cell lung cancer (NSCLC) with a poor prognosis. According to relevant research statistics, at the time of initial diagnosis, the incidence of BMs in patients with NSCLC is about 12.8%, and this proportion might rise to 25.6% in patients with advanced NSCLC. The median survival of patients with NSCLC is only 7 months (1). Current treatments for BMs typically include surgery (in selected cases for tissue diagnosis, brain decompression, and prolongation of survival), radiation therapy alone, and/or some combinations of systemic drug treatments. Radiotherapy (RT) is still the standard treatment for patients with BMs from NSCLC. However, due to the limitation of radiotherapy, the median survival time is not optimistic, and the median survival time of RPA(recursive partitioning analysis) grade III patients is only 2.3 months (2). Either whole brain radiotherapy (WBRT) or stereotactic radiosurgery (SRS) has certain limitations and adverse effects (3, 4). Therefore, there is an urgent need for optimal treatment for patients with BMs from NSCLC.

In recent years, advances in genomics have led to the development of targeted therapies for NSCLC with specific mutations. Targeted drugs represented by EGFR-TKI significantly improve the survival and prognosis of lung adenocarcinoma (5). However, in patients with advanced lung squamous cell carcinoma, the incidence of EGFR mutation and ALK gene rearrangement is only 2.7% and 1.5-2.5% (6). The benefit of TKI-targeted drug therapy is very limited, which makes it more urgent to explore the ideal treatment plan for patients with wild-type NSCLC. At present, ICIs have achieved certain safety and efficacy in the treatment of patients with wild-type NSCLC. Due to the existence of the “blood-brain barrier”, the role of anti-tumor drugs is generally ignored. Although lymphocytes in the ICIs setting of the normal brain parenchyma and primary central nervous system (CNS) tumors are rare, tumor-infiltrating lymphocytes (TILs) are prominent in BMs. Besides, the density of TILs correlates with PFS and OS in solid tumors, so the consistency of higher TILs density and improved OS supports the use of ICIs for the treatment of systemic and central metastatic disease (7). Several clinical trials have achieved encouraging results. CHECKMATE017 and CHECKMATE057showed that some patients with BMs have significantly improved OS with nivolumab (8).

There are data to suggest that the combination of ICIs and RT may further improve the status of patients with BMs. Many mechanisms have been used to explain this combined effect, such as the indirect modulation of radiation for the expression levels of immune checkpoint on the surface of cancer cells and immune cells in the tumor microenvironment through interferon-γ. A recent study showed that radiation-induced DNA double-strand breaks upregulate Programmed cell death ligand protein-1(PD-L1) expression on tumor cells *via* ATM/AR/Chk1 kinases (9). Abdulhaleem et al. published a series of studies about patients with BMs from NSCLC. If these patients were treated with ICIs and SRS, their median survival was 40 months, and if they were treated with SRS alone, their median survival was 8 months. Therefore, RT combined with ICIs may be a favorable treatment option for patients with BMs. However, there is currently no large-sample randomized controlled trial data on ICIs combined with RT, and there is still some controversy. In addition, chemotherapy has been reported to benefit patients with BMs by simultaneously treating both primary cancer and BMs. Studies have shown that compared with WBRT alone, temozolomide (TMZ) combined with WBRT in the treatment of patients with BMs from NSCLC has a higher effective rate and longer progression-free survival (10). But other chemotherapeutic drugs generally were not with the ability to cross the blood-brain barrier and reach the targeted lesion. Therefore, there is a certain controversy in the treatment of patients with BMs by chemotherapy.

In conclusion, although RT is the most important treatment for patients with BMs from NSCLC, it is necessary to explore the RT-based combination therapy to prolong the survival of patients, especially for patients without targeted epidermal growth factor receptor (EGFR) mutations/anaplastic lymphoma kinase (ALK) rearrangement. At present, a large number of studies on RT-based combination therapy (such as chemotherapy, Immune checkpoint inhibitors, etc.) are ongoing. Some of the research results have been published, but there is still a lack of head-to-head direct comparison of the efficacy and safety of different combination therapy regimens. Based on data from randomized controlled trials and retrospective cohort studies, this study compared comprehensively and quantitatively the efficacy of RT-based combination therapy in the treatment of BMs from NSCLC with non-EGFR mutation/ALK gene rearrangement. Our use of a Bayesian network meta-analysis allows comparisons between treatments that have never been evaluated in existing trials, and provides new insights into the relative efficacy and established quality advantages.

## Methods

This study was reported according to the Preferred Reporting Items for Systematic Reviews and Meta-Analyses (PRISMA 2020) (11). Besides, this study was registered in PROSPERO (CRD:42022350065).

### Search strategy and inclusion criteria

We conducted a computerized search of PubMed, Web of Science, the Cochrane Library, and Embase, the search strategy strictly followed the Population Intervention Comparative Outcomes Study (PICOS) design framework, including the following fields of Medical Subject Heading (MeSH) terms: “NSCLC” and “RT”,MeSH and subtitles were combined with “AND” or “OR”. The language type of the included studies is English. We included studies about RT-based combination therapy for BMs from NSCLC from May 28, 2002, to February 21, 2022, i.e. articles. No articles in the databases before May 28, 2002, met the inclusion criteria. In addition, we manually searched for relevant reviews and articles with included trials for additional references. Search terms related to “brain metastases”, “ICIs”, “targeted therapy”, “RT” and “chemotherapy” were included. The full set of search terms and strategies for each database were showed in **Supplementary Table S1**.

References meeting the following criteria were included, Firstly, patients with BMs from NSCLC (in our analysis, “mutation agnostic” studies were defined as all patients with NSCLC, regardless of target mutation status. “Wild-type” studies are those that explicitly include only wild-type (no EGFR mutation/ALK rearrangement) primary patients with NSCLC. Then, comparing at least two independent treatment regimens for BMs from NSCLC; And reporting sufficient information to calculate hazard ratios (HR). References were excluded based on the following criteria: 1.Patients with definite driver gene EGFR/ALK-positive; 2.Letters and abstracts; 3.Single-arm studies; 4.Non-English literature.

### Data extraction and assessment of the risk of bias

We excluded review articles, case series, case reports, guidelines, and conference abstracts; full-text studies that met the inclusion criteria were thoroughly reviewed. Two researchers (WM, JJ) independently reviewed the full text and extracted the study type, sample size, median age of patients, percentage of male/female, treatment plan and specific interventions (including specific methods and doses of radiotherapy), median follow-up time, outcome measures (OS, PFS, iPFS, grade 3/4 adverse events), medians of OS, iPFS and PFS, and the median number of BMs to an electronic database. Any differences among researchers were resolved through discussion and consensus. The risk of bias was assessed by tools from the Cochrane Collaboration (11), and other trials were assessed by Risk If Bias in non-randomized intervention studies (Robins-I) (12).

### Data synthesis and analysis

Our study endpoints were intracranial progression-free survival (iPFS), overall survival (OS), overall progression-free survival (PFS), and grade 3/4 adverse events. iPFS is generally considered to be the median survival time without radiographic intracranial progression or death from any cause (13). Because the number of analyzable co-adverse events from grade 3/4 adverse events was insufficient for statistical analysis, we analyzed only OS, PFS, and iPFS separately, and reported each outcome in the appropriate network. quantitatively only studies reporting comparisons of hazard ratios between interventions were used in the analysis, all other studies were reported qualitatively. Results of OS in analysis were expressed as a HR with a confidence interval (CI) of 95%. *P*<0.05 was considered a significant level. Heterogeneity was assessed with the I2 statistic. I2 values less than 25% and greater than 50% were considered to be low and high heterogeneity, respectively.

Only studies reporting comparisons of HR between interventions were used in our quantitative analysis, all other studies reported qualitatively. Results of OS in Bayesian network meta-analysis were expressed as a HR with a confidence interval (CI) of 95%. P<0.05 was considered a significant level. Heterogeneity was assessed with the I^2^ statistic. I^2^ values less than 25% and greater than 50% were considered to be low and high heterogeneity, respectively (14). When included studies did not report HRs, we estimated them from summary statistics using the method described by Tierney et al. in 2007 (12). We used Getdata Graph Digiamer2.26 (http://www.getdata-graphdigitizer.com) to digitize the Kaplan-Meier curve. We used GeMTC version 0.14.3 (http://drugis.org/software/addis1/gemtc) and employed a random response model for Bayesian network meta-analysis. The parameters of the GeMTC software were chosen as tuning iterations, 20,000; simulation iterations, 50,000. We ranked outcome of the five treatments (RT alone, ICIs alone, RT combined with chemotherapy, RT combined with ICIs, RT combined with targeted therapy) from the best (rank 1) to the worst (rank 5) using the ranking probabilities calculated by the network-consistent model. The rank probability distribution of each treatment was plotted in a histogram.

The histograms showed the ranking probability distribution of each treatment at each possible position. We evaluated the convergence of the model using the potential scale reduction factor (PSRF) of the Brooks-Gelman-Rubin method (13). The closer the PSRF is to 1, the better the convergence of the model. We converted the data format and used STATASE15 software to draw network diagrams and funnel plots to determine whether there was publication bias. As the network diagram did not form a closed loop, the node splitting method was not examined.

## Results

### Baseline characteristics of the included studies

**Figure 1:**
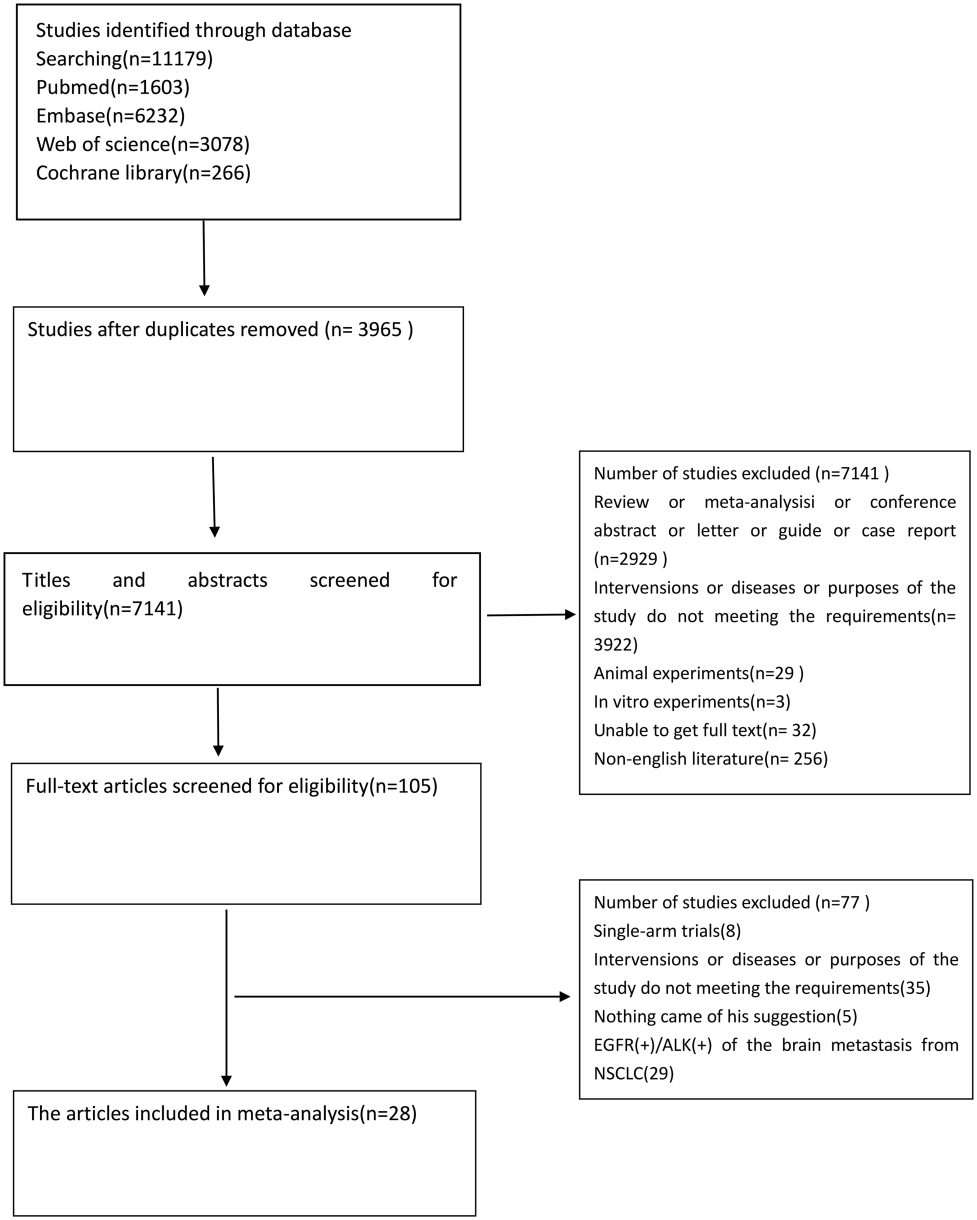
Flow diagram for the selection of the papers.

We identified 11,179 studies by searching the databases (**Figure 1**). Duplications were removed, and 7,141 papers were for the title and abstract screening. After excluding studies, such as conference abstracts, non-English papers, and non-related interventions, 28 papers, including 12 randomized controlled trials, were finally included in the Bayesian network meta-analysis. A total of 3703 patients received at least one of the five treatment strategies (**Table 1**) (15–42).

**Table 1:** Characteristics of the included studies.

Study	Type	Patient no.(T/C)	Mean ages(years)	Female(T/C%)	Intervention	Details	Median Follow-up (months)	outcomes	The median numbers of Brain metastases	Primary CancerType (No.)	Medians of OS, iPFS and PFS (months)
Guo et al., 2022 (15)	Cohort	26/84	57.8	19.2/26.2	ICI+RT/ICI	WBRT 30~40gy/10~20f	13.2	OS;PFS	2	ALL NSCLC	OS:25.4/14.6 iPFS:9.3/4.2 PFS:4.3/2.7
Abdulhaleem et al., 2022 (16)	Cohort	80/235	64	60.0/46.8	ICI+RT/RT	Pembrolizumab;Nivolumab;Atezolizumab;Durvalumab; SRS:18.6~19.4gy/1f	NA	OS	NA	ALL NSCLC	OS:40/8 iPFS : NA PFS : NA
Scoccianti et al., 2021 (17)	Cohort	100/50	64	37/38	ICI+RT/RT	SRT 28.99gy/3f Nivolumab PembrolizumabAtezolizumab	23	iDFS;ipfs;os	2	ALL NSCLC	NA
Samuel et al., 2021 (18)	Cohort	102/167	70	25/37	ICI+RT/ICI	Nivolumab3mg/kg;Pembrolizumab 200mg; WBRT/SRS/WBRTplusSRS 20gy/5f	19.4	PFS;OS;ORR;DCR	NA	ALL NSCLC	OS:9/9 iPFS : NA PFS:3/2
Metro et al., 2021 (19)	Cohort	8/9	66.4	62.5/55.6	ICI+RT/ICI	Pembrolizumab 200mg/3weeks SRS;WBRT	10.2	PFS, TTF, BPFS, OS	NA	ALL NSCLC	OS : NA iPFS : NA PFS:7.1/10.2
Lu et al., 2021 (20)	Cohort	21/28	57	33/42	TT+RT/RT	Bevacizumab 5~7.5mg/kg WBRT-SRS 3gy/10f	13.53	ORR;DCR;OS;LPFS	NA	ALL NSCLC	OS:42.63/25.23 iPFS:39.53/23 PFS : NA
Liao et al., 2021 (21)	Cohort	29/41	58.4	31/69	ICI+RT/RT	WBRT 30gy/10f	17	PFS;OS;ODS	NA	ALL NSCLC	OS:27/20 iPFS : NA PFS:12/7
Lee et al., 2021 (22)	Cohort	51/26	60	49/34	ICI+RT/ICI	Pembrolizumab 2 mg/kg/3 weeks; Nivolumab 3 mg/kg/3 weeks; GKS 19gy/f	19.1	iPFS;L-PFS;LMS-PFS;OS	2	ALL NSCLC	OS:42.1/10 iPFS:7.9/3.4 PFS:11.5/NA
Khan et al., 2021 (23)	Cohort	10/11	56	17/83	ICI+RT/RT	WBRT 30gy/10f	13	OS;PFS	NA	ALL NSCLC	OS:24/13 iPFS : NA PFS:11/3
He et al., 2021 (24)	Cohort	28/45	58.5	46.43/46.67	TT+RT/RT	Anlotinib 8~12mg/kg; CRT 30~40gy/10~20f; LCRT 25~54gy/5~27f; WBRTplusLCRT 30~40gy/fplus10~24gy/f	8	ORR;OS;PFS;IPF;EPF;SPF; iPFS;ePFS	NA	ALL NSCLC	OS:8.5/6 iPFS:11/3 PFS : NA
Enright et al., 2021 (25)	Cohort	33/44	62.7	39.4/38.6	ICI+RT/RT	Atezolizumab Durvalumab Nivolumab Pembrolizumab SRT 25gy/5f	11.4	OS;LC;DBF	2	ALL NSCLC	NA
Guenole et al., 2020 (26)	Cohort	30/95	60.6	49/38	ICI+RT/RT	Nivolumab 3 mg/kg/2 weeks; Pembrolizumab 2 mg/kg/3 weeks; Durvalumab 10 mg/kg/2 weeks; Ipilimumab 3 mg/kg/3 weeks;SRT 21~23.1gy/y	11.9	OS;HR;OR	2	NA	NA
Shepard et al., 2019 (27)	Cohort	17/34	64.2	35.3/41.2	ICI+RT/RT	SRS 18.4 ± 2.3gy/1f	10	PFS;OS	2.7	ALL NSCLC	OS:15.9/NA iPFS:6.6/NA PFS : NA
Lanier et al., 2019 (28)	Cohort	101/170	66.4	46/45	ICI+RT/RT	Nivolumab.Pembrolizumab Ipilimumab.Atezolizumab. SRS 18gy/y	29.9	OS	NA	Lung cancer (226), other (45)	OS:15.9/6.1 iPFS : NA PFS : NA
Chen et al., 2018 (29)	Cohort	79/181	NA	NA	ICI+RT/RT	SRS/SRT 15~24gy/1f or 18~24gy/3f or 25gy/5f; Nivolumab;Pembrolizumab;lpilimumab	9.2	PFS;OS	2	NSCLC (157), other (103)	OS:24.7/12.9 iPFS : NA PFS : NA
Deng et al., 2017 (30)	Cohort	129/109	60	46.5/38.5	CT+RT/RT	Temozolomide 75mg/m^2^;100mg/m^2^ WBRT 3gy/10f	NA	ORR;DCR;OS;iPFS	NA	ALL NSCLC	OS:8.5/5.9 iPFS:5.9/4.9 PFS : NA
Chabot et al., 2017 (31)	RCT	103/102	60	38/45	TT+RT/RT	Veliparib 50mg bid or 200mg bid;WBRT 30gy/3gy/10f	36	OS	NA	ALL NSCLC	OS:7/6.2 iPFS : NA PFS : NA
Lim et al., 2015 (32)	RCT	49/49	57.9	29/27	CT+RT/RT	Cisplatin 60mg/m^2^ plus Gemcitabine 1000mg/m^2^; Cisplatin 70mg/m^2^ plus Pemetrexed 500mg/m^2^/Docetaxel 75mg/m^2^; Cisplatin 60mg/m^2^ plus Etposide 100mg/m^2^;SRS	43	OS;PFS;ORR	NA	ALL NSCLC	OS:15.3/14.6 iPFS:9.4/6.6 PFS : NA
Lee et al., 2014 (33)	RCT	40/40	61.2	62.5/47.5	TT+RT/RT	WBRT 20gy/5f;Erlotinib 150mg	12.6	PFS;OS	NA	ALL NSCLC	OS:3.4/2.9 iPFS : NA PFS : NA
Sperduto et al., 2013 (34)	RCT	41/44	NA	NA	TT+RT/RT	WBRT 37.5gy/2.5gy/15f; Erlotinib 150mg/d;	33.6	OS	NA	ALL NSCLC	OS : NA iPFS:4.8/8.1 PFS : NA
Sperduto et al., 2013 (34)	RCT	40/44	NA	NA	CT+RT/RT	WBRT 37.5gy/2.5gy/15f; Temozolomide 75mg/m^2^;/d;	33.6	OS	NA	ALL NSCLC	OS : NA iPFS:4.6/8.1 PFS : NA
Hassler et al., 2013 (35)	RCT	22/13	65	41/38.5	CT+RT/RT	Temozolomide75mg/m^2^;WBRT 3gy/10f;2gy/20f	NA	TTP;OS;PFS	NA	ALL NSCLC	OS:3/6.3 iPFS : NA PFS : NA
Ge et al., 2013 (36)	Prospective	38/38	58	36.8/39.5	CT+RT/RT	Topotecan1.75mg/m^2^;RT2gy/5f;DT 40gy/20f	36	PFS;OS	NA	ALL NSCLC	OS:13/10 iPFS : NA PFS:6/3
Groberg et al., 2012 (37)	RCT	54/53	62.7	43.6/37	CT+RT/RT	Ezastaurin 375mg tid;WBRT 4gy/5f or 5gy/4f or 3gy/10f	NA	OS;PFS;ORR;TTP	NA	NSCLC (80), other (29)	OS:3.8/5.1 iPFS : NA PFS:2.2/2
Chua et al., 2010 (38)	RCT	47/48	60	36/33	CT+RT/RT	WBRT 30gy/10f;Temozolomid 75mg/m^2^	24	OS;PFS	NA	ALL NSCLC	OS:4.4/5.7 iPFS:3.1/3.8 PFS : NA
Neuhaus et al., 2009 (39)	RCT	47/49	57.8	31.9/38.8	CT+RT/RT	WBRT 2gy~40gy/5f Topotecan 0.4mg/m^2^	34	PFS	NA	ALL NSCLC	OS:2.9/3.2 iPFS : NA PFS:2.4/2.2
Verger et al., 2005 (40)	RCT	41/41	58.1	66/63	CT+RT/RT	Temozolomid75mg/m^2^/d or 200mg/m^2^/d;WBRT 30gy/3gy/10f	NA	PFS;OS	2	ALL NSCLC	NA
Guerrieri et al., 2004 (41)	RCT	21/21	61	28.6/28.6	CT+RT/RT	Carboplatin 70mg/m^2^ WBRT 20gy/5f	NA	OS;ORR	NA	ALL NSCLC	OS:3.7/4.4 iPFS : NA PFS : NA
Antonadou et al., 2002 (42)	RCT	25/23	NA	24/30.4	CT+RT/RT	Temozolomid 75mg/m^2^/d;WBRT 40gy/4f	4	ORR	NA	NSCLC (31), other (17)	NA

CT+RT, chemotherapy combined with radiotherapy; ICI, Immune checkpoint inhibitor; ICI+RT, Immune checkpoint inhibitor combined with radiotherapy; RT, radiotherapy; TT+RT, targeted therapy combined with radiotherapy; WBRT, whole-brain radiation therapy; SRS, stereotactic radiosurgery; SRT, stereotactic radio therapy; GKS, Gamma Knife Radiosurgery; CRT, conformal radiation therapy, LCRT, local conformal radiation therapy; OS, overall survival; PFS, progression-free survival, iPFS, intracranial progression-free survival; TTF, time-to-treatment failure; LC, local control; DBF, distant brain failure; TTP, Median time to progression, ORR, overall response rate, DCR, disease control rate; NSCLC, none-small cell lung cancer; NA, not applicable.

**Figure 2:**
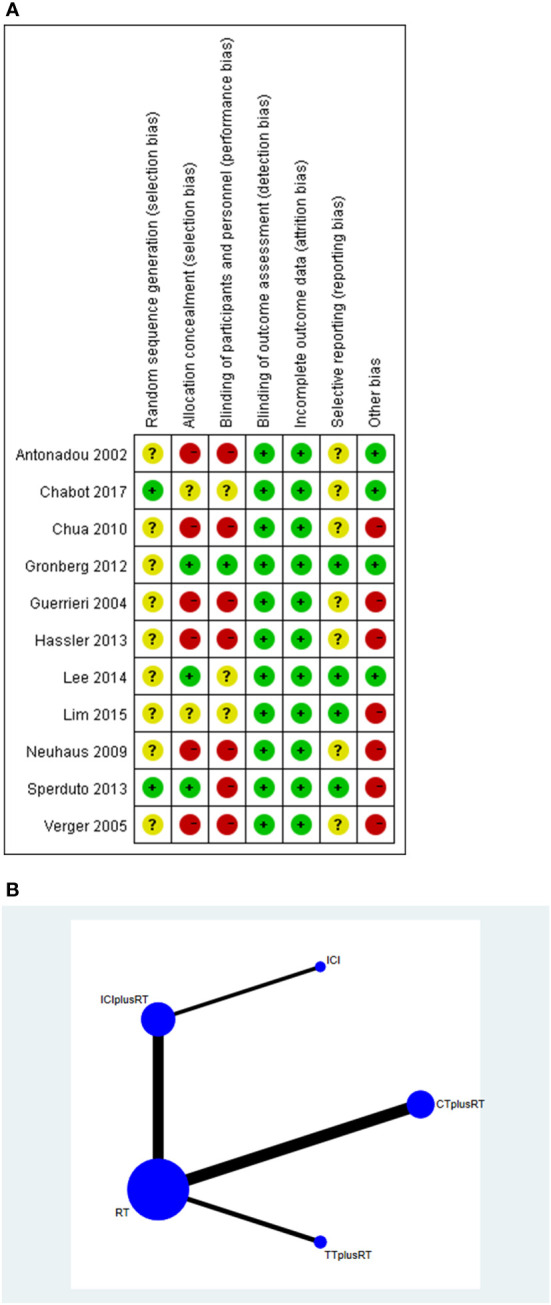
**(A)** risk of bias summary, green represents low risk of bias, yellow represents unclear risk of bias, red represents high risk of bias; **(B)** network.

All eligible studies were published from 2002 to 2022. We used the Cochrane Collaboration tool and Risk If Bias in a non-randomized intervention study (Robins-I) for quality assessment. the results of the quality assessment were shown in **Figure 2A** and **Table 2**. **Figure 2A** | The reviewers judged the risk of bias for each included study, and 6 of the 12 studies were open-label trials (35, 38–42), without blinding in study design. 6 studies recruited less than expected (32, 34, 35, 39–41), 9 studies did not mention random sequence generation (32, 33, 35, 37–42), 3 studies did not mention study blinding design (31–33), and other aspects were assessed as high quality. **Table 2** | Of the cohort studies, 12 studies did not specify whether subjects had developed the focused disease (15, 16, 18, 20, 23, 24, 26–30, 36), and were rated as high risk, 25 studies only mentioned part follow-up related data (15–21, 23, 25–30), which is not enough to judge the completeness of the follow-up data, and were rated as high risk, and the rest included studies were low risk. We rated articles with a score of ≥ 6 as high quality, and all included studies were high quality.

**Table 2:** Cohort study quality assessment.

Questi/n/Studies	Is the exposed cohort trulyrepresentative of the average?	Wasthe selection of nonexposed cohort derived from the samecommunity as the exposed corhort?	Did the ascertainment of exposure come from secure records?	Was the outcome of interest not present at start on study?	Is the study starting with no subjects when the disease under study has occurred?	Was the follow-up longenough for outcomes to occur?	Is the result reliable?	Was the follow up of coh0rts adequate(subjects lost to follow-up<10%)	Sum
Guo et al., 2022 (15)	×	×	×	×	/	×	×	/	6
Abdulhaleem et al., 2022 (16)	×	×	×	×	/	×	×	/	6
Scoccianti et al., 2021 (17)	×	×	×	×	×	×	×	/	7
Samuel et al., 2021 (18)	×	×	×	×	/	×	×	/	6
Metro et al., 2021 (19)	×	×	×	×	×	×	×	/	7
Lu et al., 2021 (20)	×	×	×	×	/	×	×	/	6
Liao et al., 2021 (21)	×	×	×	×	×	×	×	/	7
Lee et al., 2021 (22)	×	×	×	×	×	×	×	×	8
Khan et.al. 2021 (23)	×	×	×	×	/	×	×	/	6
He et al., 2021 (24)	×	×	×	×	/	×	×	×	7
Enright et al., 2021 (25)	×	×	×	×	×	×	×	/	7
Guenole et.al. 2020 (26)	×	×	×	×	/	×	×	/	6
Shepard et.al. 2019 (27)	×	×	×	×	/	×	×	/	6
Lanier et.al. 2019 (28)	×	×	×	×	/	×	×	/	6
Chen et.al. 2018 (29)	×	×	×	×	/	×	×	/	6
Deng et al., 2017 (30)	×	×	×	×	/	×	×	/	7
Ge et al., 2013 (36)	×	×	×	×	/	×	×	×	7

“×” represents yes; “/” represents no.

Our study compared five interventions: RT combined with ICIs, RT combined with targeted therapy, RT combined with chemotherapy, RT alone, and ICIs alone. The network was shown in **Figure 2B**. The thickness of each line in the network diagram is proportional to the number of comparisons. Based on DIC values, random-effects models were applied to the PSA-PFS, time to SSE, and OS in the Gleason Score ≥8 subgroups analysis; fixed-effects models were applied to other comparisons.

### OS

**Figure 3:**
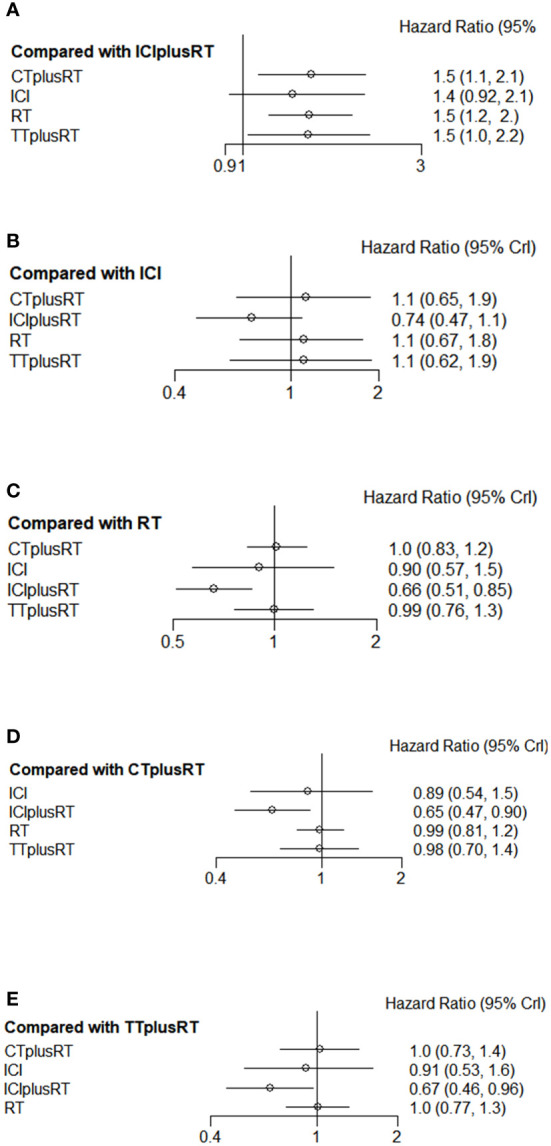
Forest plots of multivariable interventions for OS. **(A)** Compared with Immune checkpoint inhibitor combined with radiotherapy (ICIplusRT); **(B)** Compared with Immune checkpoint inhibitor (ICI); **(C)** Compared with radiotherapy (RT); **(D)** Compared with chemotherapy combined with radiotherapy(CTplusRT); **(E)** Compared with targeted therapy combined with radiotherapy (TTplusRT).

**Figure 4:**
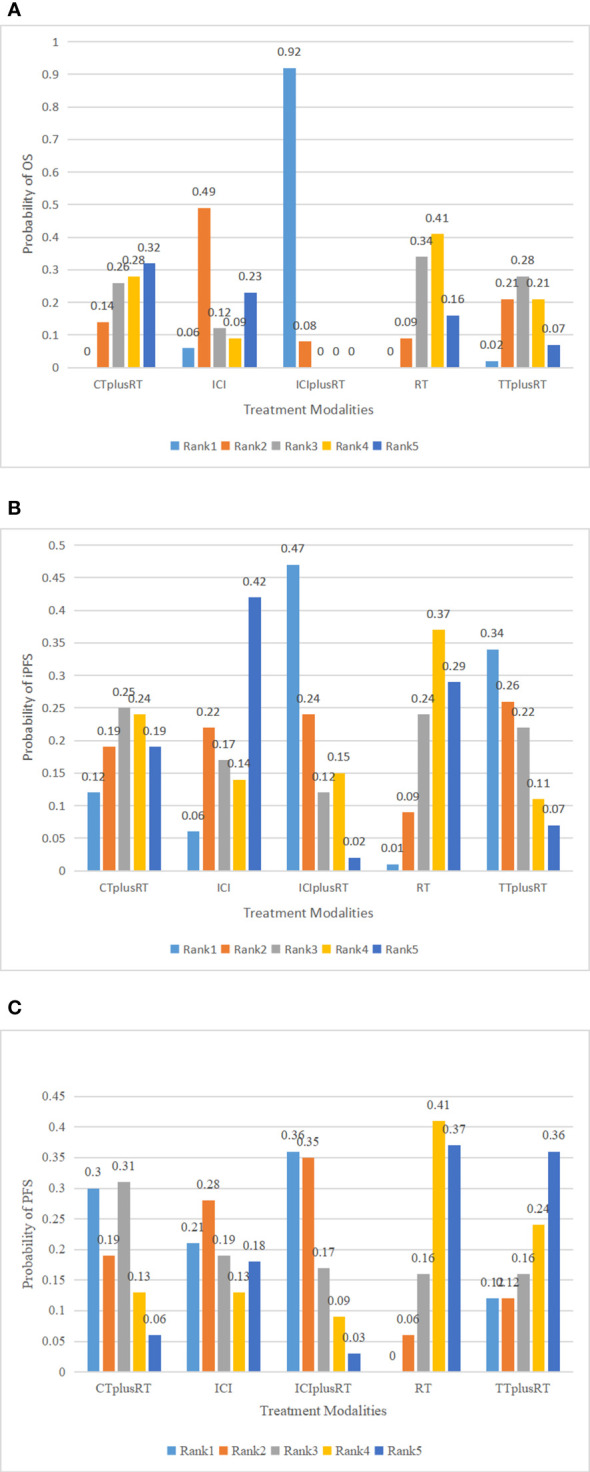
Ranking of treatments in terms of overall survival (OS), progression-free survival (PFS), and intracranial progression-free survival (iPFS). Abbreviations: CT+RT, chemotherapy combined with radiotherapy; ICI, Immune checkpoint inhibitor; ICI+RT, Immune checkpoint inhibitor combined with radiotherapy; RT, radiotherapy; TT+RT, targeted therapy combined with radiotherapy; **(A)** ranking of treatments to OS; **(B)** ranking of treatments to iPFS; **(C)** ranking of treatments to PFS.

A total of 28 studies were included in the OS analysis, and ICIs+RT (HR=0.65, 95% confidence interval: 0.47-0.9) had a survival benefit over CT+RT; ICIs+RT (HR=0.66, 95% confidence interval: 0.51-0.85) had a survival benefit over RT alone; ICIs+RT (HR=0.67, 95% confidence interval: 0.46-0.96) had a survival benefit over TT+RT alone. The other interventions were not statistically significant. ICIs+RT was the most effective combination regimen (92%), while the possibility of TT+RT (29%) was the lowest. The pooled HR for OS were shown in **Figure 3A-E** and the detailed ranking results were shown in **Figure 4A**.

### iPFS

**Figure 5:**
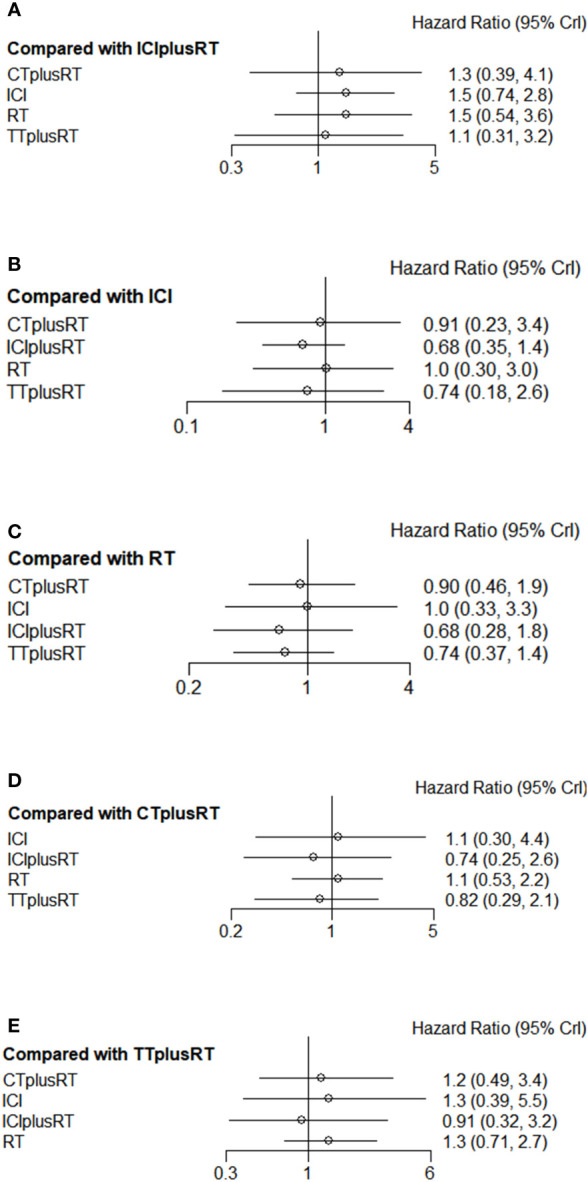
Forest plots of multivariable interventions for iPFS. **(A)** Compared with Immune checkpoint inhibitor combined with radiotherapy (ICIplusRT); **(B)** Compared with Immune checkpoint inhibitor (ICI); **(C)** Compared with radiotherapy (RT); **(D)** Compared with chemotherapy combined with radiotherapy(CTplusRT); **(E)** Compared with targeted therapy combined with radiotherapy (TTplusRT).

Ten studies were included in the iPFS analysis, and there was no statistical significance in the indirect pairwise comparison of the five treatments. In the ranking, ICIs+RT was the most effective combination treatment (45.3%), while ICIs (43.8%) ranked last. The pooled HR for iPFS were shown in **Figure 5A-E**, and the detailed ranking results were shown in **Figure 4B**.

### PFS

**Figure 6:**
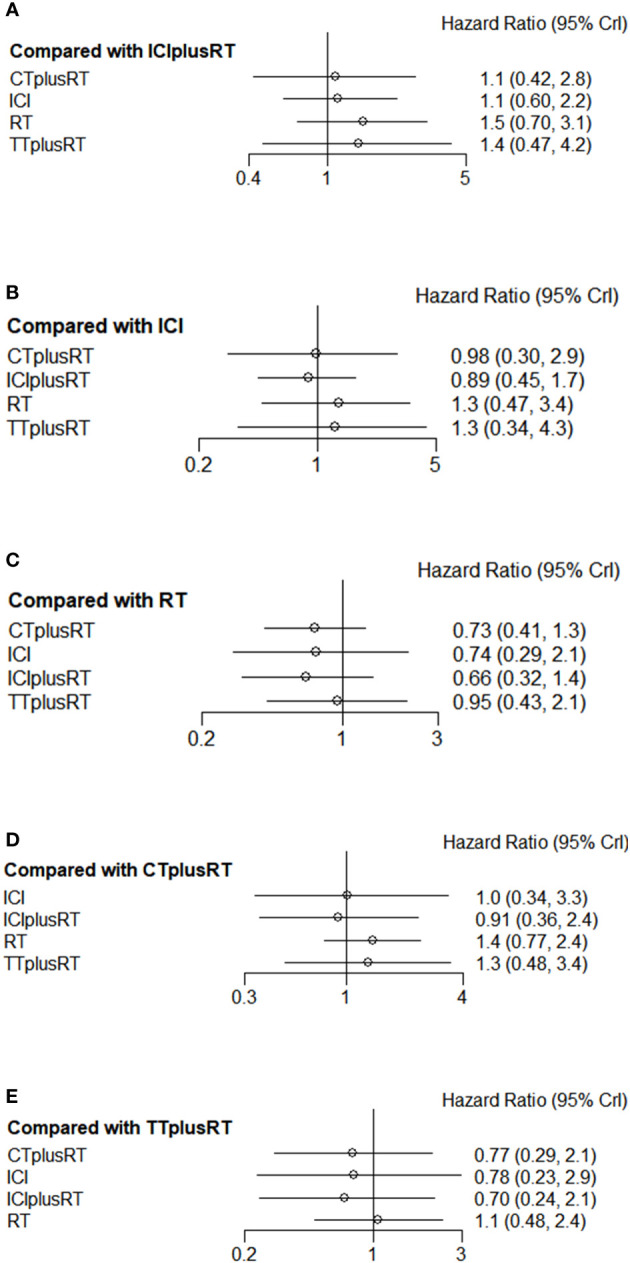
Forest plots of multivariable interventions for PFS. **(A)**Compared with Immune checkpoint inhibitor combined with radiotherapy (ICIplusRT); **(B)** Compared with Immune checkpoint inhibitor (ICI); **(C)** Compared with radiotherapy (RT); **(D)** Compared with chemotherapy combined with radiotherapy(CTplusRT); **(E)** Compared with targeted therapy combined with radiotherapy (TTplusRT).

Twelve studies were included in the PFS analysis, and there was no statistical significance in the indirect pairwise comparison of the 5 treatments. In the ranking, ICIs+RT was the most effective combination regimen (36%), while TT+RT (36.1%) ranked last. The pooled HR for PFS were shown in **Figure 6A-E**, and the detailed ranking results were shown in **Figure 4C**.

### 3/4 grade adverse effects

All included studies reported adverse effects. 10 studies reported no 3/4 grade or higher adverse effects (15, 20, 22–27), and the remaining 18 studies reported 839 adverse events, The ICIs combined RT intervention accounted for 65 cases. The reported adverse effects involved different systems and symptoms. The most common adverse effects were on the gastrointestinal tract and CNS. Details of the reported safety concerns were provided in **Table 3**.

**Table 3:** Adverse event.

English ID	AE of injective group	AE of control group
Guo et al., 2022 (15)	none	none
Abdulhaleem et al., 2022 (16)	radiation effect (2)	radiation effect(2)
Scoccianti et al., 2021 (17)	radionecrosis (1)	None
Samuel et al., 2021 (18)	colitis(2),pneumonitis(2),dermatitis(2),pancreatitis(1),polymyositis(3)	colitis(1),pneumonitis(3),dermatitis(2),nephritis(2),gastritis(1),encephalitis(1),polymyositis(1)
Metro et al., 2021 (19)	colitis(1), skin rash(1), mucositis(1)	pancreatitis(1)
Lu et al., 2021 (20)	none	none
Liao et al., 2021 (21)	nausea/vomiting(1), hyponatremia(1)	none
Lee et al., 2021 (22)	none	none
Khan et.al. 2021 (23)	none	none
He et al., 2021 (24)	none	none
Enright et al., 2021 (25)	none	none
Guenole et.al. 2020 (26)	none	none
Shepard et.al. 2019 (27)	none	none
Lanier et.al. 2019 (28)	CNS toxicity(21)	none
Chen et.al. 2018 (29)	acute CNS toxicity(1),immune-related adverse event(7)	acute CNS toxicity(7)
Deng et al., 2017 (30)	fatigue(16),anorexia(14),nausea(29),vomiting(14),headache(13),anemia(5),neutropenia(13),thrombocytopenia(4)	fatigue(12),anorexia(9),nausea(20),vomiting(13),headache(11),anemia(3), neutropenia(10),thrombocytopenia(2)
Chabot et al., 2017 (31)	pneumonia(3),fatigue(2),pain(1), anemia(1),malignant neoplasm progression(2),pulmonaryembolism(4), thrombocytopenia(3), hyperglyccmia(2)	pneumonia(6),fatigue(4), pain(4),anemia(3), dehydration(3),brainedema(3),convulsion(3), malignant neoplasm progression(2),pulmonary embolism(1),thrombocytopenia(1), hyperglyccmia(1)
Lim et al., 2015 (32)	none	none
Lee et al., 2021 (22)	dyspnoea(14),fatigue(7),rash(8), infection(5),myopathy(2),anorexia(2), pain(2),diarrhoea(2),dehydration(2),pulmonary(1),somnolence(1),constipation(1),dry shin(1),nausea(1)	dyspnea(15),fatigue(14),rash(2),infection(2), myopathy(4),anorexia(3), pain(3),diarrhoea(2), headache(4),muscle weakness(3),anaemia(2),casepulmonary(1)embolism(1),seizure(2),somnolence(1), pneumonitis(1)
Sperduto et al., 2013 (34)	cytopenia, fatigue, dehydration, gastrointestinal bleeding, infection, hyperglycemia, seizures,cytopenia, hypokalemia,fatigue, thrombocytopenia(all:16)	anemia, fatigue, muscle weakness, confusion, headache(all:5)
Sperduto et al., 2013 (34)	cytopenia, fatigue, dehydration, acne, anorexia, vasculitis, diarrhea, pneumonia, hyperkalemia, muscle weakness, confusion,ataxia,myocardial ischemia, brain necrosis, hemorrhagic stroke.(all:20)	anemia, fatigue, muscle weakness, confusion, headache(all:5)
Hassler et al., 2013 (35)	Haematological toxicity(10),nausea and vomiting(4),consciousness disturbance(5),coordination(6),mood disturbance(6),change of behaviour(2),vertigo(2),sleep disturbance(5)	Haematological toxicity(4),nausea and vomiting(1),consciousness disturbance(1),mood disturbance(1),change of behaviour(1),vertigo(1),sleep disturbance(3)
Ge et al., 2013 (36)	leukopenia(2),neutropenia(2),thrombocytopenia(1)	none
Groberg et al., 2012 (37)	platelets(3),neutrophils(3),fatigue(9),thrombosis/thrombus/embolism(7),lung infection(5),nausea(7),motor neuropathy(4),dyspnea(3),vomiting(3)	platelets(1),fatigue(5),thrombosis/thrombus/embolism(9),lung infection(6),nausea(3),motor neuropathy(1),dyspnea(4),vom-iting(2)
Chua et al., 2010 (38)	deep vein thrombosis(1),chest pain and dyspnea(1),sudden death(1), hematology and bloodchemistry(15)	hematology and blood chemistry(9)
Neuhaus et al., 2009 (39)	granulocytes(4),haemoglobin(2), leukocytes(7),thrombocytes(11),alopecia(20),infection(12),somnolence(2),dyspnea(5),4nausea(4),vomiting(1),constipation(5),pain(7),stomatitis(3),hyperglycaemia(2),gihaemorrhage(1),sensorium(3), creatinin-elevated(2),pneumonitis(1)	granulocytes(1),alopecia(19),infection(6),somnolence(3),dyspnea(1),nausea(3), constipation(3),pain(12),stomatitis(6),hyperglycaemia(1), gi-haemorrhage(1), sensorium(1),creatinin-elevated(3),pneumonitis(3)
Verger et al., 2005 (40)	nausea andvomiting(1),neutropenia(5), thrombocytopenia(4)	none
Guerrieri et al., 2004 (41)	none	none
Antonadou et al., 2002 (42)	headache(10),nausea(12),vomiting(8),fatigue(9)	headache(6),nausea(3),fatigue(7)

CNS, central nervous system.

### Convergence, inconsistency, publication bias, and heterogeneity analysis

The potential scaling factor was limited to 1, reflecting the good convergence of this study. The funnel plots of included trials were nearly symmetrical, suggesting no apparent publication bias. Considering that there were no closed loops in the network graph, inconsistency evaluation did not apply to our study. The OS heterogeneity analysis of the entire network showed that the value of RT alone versus ICIs combined with RT was 70.1%, and the value of ICIs alone versus ICIs combined with RT was 79.7%. There was high heterogeneity. This may be related to the inclusion of patients with different pathological tumor types than NSCLC. The results of convergence, inconsistency, publication bias, and heterogeneity can be found in the **Supplementary Figures**.

## Discussion

We conducted a Bayesian network meta-analysis of the efficacy of RT-based combination therapy for BMs from NSCLC with non-EGFR mutation/ALK gene rearrangement. The result showed that, compared with RT, RT combined with chemotherapy and RT combined with target therapy, ICIs combined with RT had a significant OS benefit, regardless of whether OS was counted from the date of diagnosis of BMs or the date of RT. In terms of iPFS and PFS, ICIs combined with RT was also the most effective treatment option, with moderate to high certainty. There were no significant differences in grade 3/4 adverse effects between the ICIs combined with the RT group and the other treatment groups, indicating that ICIs combined with RT was tolerable.

In the era of immunotherapy, the anti-PD-1 antibody Pembrolizumab has been approved as a first-line treatment for PD-L1-positive advanced NSCLC (43), and related mechanisms also support the efficacy of ICIs in patients with BMs from NSCLC. After immunotherapy, the vascular permeability of lymphocytes increases, and a large number of activated T lymphocytes derived from the primary tumor and deep external cervical lymph node tissue penetrate the blood-brain barrier to exert intracranial antitumor activity (44).

In the study by Teixeira et al. (45), comparing ICIs alone with ICIs+RT, no intracranial disease control rate (iDCR) and objective response rate (iORR) were observed in patients with BMs who received RT before the initiation of ICIs. There was a statistical difference between patients with BMs who received RT before ICIs and those who received ICIs alone. Considering radiation necrosis, ICIs alone should be considered the first-choice treatment for patients with active NSCLC with BMs. The above is inconsistent with our conclusions. There may be the following reasons. First, in terms of the sample size of the included population, they only included 566 people, which is much smaller than ours. Then, there is no restriction on the sequence of RT combined ICIs in our study. And the main outcome of our meta-analysis was OS, iPFS, and PFS, while Teixeira’s study did not perform statistical analysis from survival indicators due to not enough data. Finally, the two articles included patients with inconsistent brain metastases, and our study included a population with stable BMs at baseline. In the Keynote-042 study (46), pembrolizumab worked only in patients with untreated or brain metastases 5 to 20 mm in diameter. In addition, the use of ICIs alone in the treatment of patients with BMs from NSCLC is controversial. We analyzed ICIs combined with RT in the treatment of patients with BMs and found that the improvement in OS may be largely due to RT can promote the anti-tumor efficacy (44) of ICIs by inducing T lymphocytes to release tumor antigens and activate antigens. In the study by Kim et al. (47), the local response rate (ORR) of ICIs combined with RT was superior to ICIs monotherapy. There was no difference in the incidence of grade 3/4 CNS related adverse events (5% vs 4%; p=0.93). Compared with ICIs monotherapy, patients treated with the combination of ICIs and RT had better overall survival and intracranial progression-free survival. In addition, in the study by Yang et al. (48), the overall survival (OS) of brain RT combined with ICIs was significantly better than that of brain RT alone compared with the brain RT alone group. In the treatment of patients with NSCLC BMs, RT combined with ICIs has better efficacy. From the studies we included, it can be seen that when combined therapy is given, radiotherapy is mostly SRS and WBRT, with a few studies using stereotactic radiosurgery (SRT) and gamma knife surgery (GKS). The ICIs involved in the studies mainly include Nivolumab, Pembrolizumab, Atezolizumab, Durvalumab, etc. The available evidence suggests that simultaneous combination of ICIs with SRS, Kotecha et al. (49) enrolled 150 BM patients and found that the group receiving SRS in combination with ICIs had a higher objective remission rate than the SRS group alone, and a subgroup analysis concluded that the combination was most effective within one ICIs half-life before and after SRS, so many studies have defined synchronous treatment as receiving RT within one month before and after ICIs, Although there is a lack of prospective high-quality evidence on the optimal timing of radiotherapy combined with immunotherapy and the specific dose of the combination, the available evidence suggests that the combination of ICIs with RT for brain metastases may improve efficacy and survival without a significant increase in radiotherapy-related toxicity, and that patients with non-EGFR mutated/ALK rearranged non-small cell lung cancer BMs with indications for intracranial radiotherapy may be treated with a combination of ICIs and radiotherapy preferably with SRS. No reduction in radiotherapy dose is recommended without clear evidence.

In our study, RT combination chemotherapy in improving OS for patients with BMs from NSCLC with non-EGFR mutation/ALK gene rearrangement was inferior to RT combined with ICIs, although concurrent chemoradiotherapy is currently the first-line guideline for the treatment of such patients (43). The conclusion emphasizes the concept of patients dying of systemic disease (refers to a disease in which multiple systems of the body are involved) and the importance of maintaining cognition for as long as possible time. However, despite the efficacy of ICIs combined with RT could prolong overall survival, it still lacks iPFS, PFS benefit because our study did not specifically differentiate between RT modality. After all, the local control of BMs is mainly achieved through brain RT (48).

Among the grade 3/4 adverse effects, because there is not enough data to support statistical analysis, the grade 3/4 adverse events involved in 28 works of literature were summarized, as shown in **Table 3**, and no evidence was found. The significant differences between the RT combined with the ICIs group and the other treatment groups further confirm the reliability of our conclusions. In the meta-analysis by Sha et al. (50), which included 51 studies (n=15,398), 35 ICIs alone (n=13,956) and 16 ICIs+RT studies (n=1,442). Results showed that grade 3-4 adverse events were similar in patients receiving ICIs plus RT and ICIs alone. The above indicated that the safety of ICIs combined with RT therapy for patients with BMs from NSCLC is acceptable.

Our meta-analysis has some limitations. First, the studies in this meta-analysis included retrospective cohort studies and randomized controlled studies, and there was bias between treatment groups. Second, the included studies had a large period, RT and ICIs, chemotherapy, and targeted drug types are confounding factors, and this deficiency may have affected the pooled effect size of the data. Finally, the sample size of the included studies was not large enough for subgroup analysis, and the median number of BMs was not high. This also causes certain deviations in judging the efficacy of drugs.

However, in the absence of published articles from prospective randomized controlled trials, there is a lack of convincing evidence to support the efficacy of ICIs combined with RT in patients with BMs from NSCLC with non-EGFR mutation/ALK gene rearrangement. Our analysis is urgently needed to provide a rationale for the design of randomized controlled trials, as well as applications to guide clinical practice.

Several ongoing trials (NCT03391869, NCT04889066, NCT04787185) investigate more detailed information, including timing and sequence of combination therapy and optimal dosing, and these further studies may provide insights into the establishment of new NSCLC brain metastases in specific settings.

In addition, the three major clinical studies of ICIs combined with chemotherapy, Keynote021 (51), Keynote189 (52), and Keynote407 (53), all included patients with stable baseline BMs. Compared with chemotherapy alone, ICIs combined with chemotherapy had significant advantages in OS, PFS, ORR, etc., and the incidence of related adverse events was not significantly different from the chemotherapy group. The enhanced intracranial efficacy of this combination therapy against BMs may depend on the penetration of the blood-brain barrier by a large number of chemotherapeutic drugs, and these cytotoxic drugs induce an active ICIs microenvironment to maximize the efficacy of ICIs (44). Our meta-analysis did not include the group of ICIs combined with chemotherapy, because there are fewer related studies were comparing ICIs combined with chemotherapy and RT in our search scope. And the data from Keynote021, Keynote189 and Keynote407 could not be used in this Bayesian network meta-analysis, because they were only included in the chemotherapy combined with immunotherapy and chemotherapy alone groups and did not share a common association with our data, such as a control group of the same type or an experimental group, Future research should focus on evaluating the efficacy of ICIs combined with RT and ICIs combined with chemotherapy sex, and direct non-inferior face-to-face comparisons.

In conclusion, according to the comprehensive evaluation of Bayesian network meta-analysis, compared with chemotherapy combined with RT and RT alone, ICIs combined with RT significantly improved the OS of patients with BMs from NSCLC, and the grade 3/4 adverse reactions were acceptable. More clinical data will be needed to further determine the long-term efficacy of ICIs combined with RT.

## Data availability statement

The original contributions presented in the study are included in the article/**Supplementary Material**. Further inquiries can be directed to the corresponding author.

## Author contributions

Conceptualization: MW. Methodology: RC, XZ. Supervision: JC. Writing-original draft: MW. Writing-review and editing: JJ, QC. All authors contributed to the article and approved the submitted version.

## Funding

This work was supported by grants from the Science and Technology Agency of Qinghai Province (Grant No. 2022-ZJ-719), Qinghai Health Committee (Grant No. 2020-wjzd-03).

## Conflict of interest

The authors declare that the research was conducted in the absence of any commercial or financial relationships that could be construed as a potential conflict of interest.

## Publisher’s note

All claims expressed in this article are solely those of the authors and do not necessarily represent those of their affiliated organizations, or those of the publisher, the editors and the reviewers. Any product that may be evaluated in this article, or claim that may be made by its manufacturer, is not guaranteed or endorsed by the publisher.

## Supplementary material

Supplementary Table 1 Search strategy
