# Supplementary material for: RT-based combination therapy for brain metastasis from NSCLC with non-EGFR mutation/ALK gene rearrangement: A network meta-analysis
Source: Front Oncol. 2022 Nov 28;12:1024833. doi: 10.3389/fonc.2022.1024833 (PMC9744133; doi:10.3389/fonc.2022.1024833)
Supplement: Supplementary Table 1 — Search strategy [file DataSheet_1.doc]

**eTable 1.** Queries used for systematic search.

| Sources | Queries |
| --- | --- |
| PubMed | **((("Carcinoma, Non-Small-Cell Lung"[Mesh]) OR (((((((((Carcinoma, Non-Small-Cell Lung[Title/Abstract]) OR (Carcinoma, Non‐Small‐Cell[Title/Abstract])) OR (Carcinoma, Non Small Cell Lung[Title/Abstract])) OR (Carcinomas, Non‐Small‐Cell Lung[Title/Abstract])) OR (Lung Carcinoma*, Non‐Small‐Cell[Title/Abstract])) OR (Non Small Cell Lung Carcinoma[Title/Abstract])) OR (Carcinoma, Non‐Small Cell Lung[Title/Abstract])) OR (Non‐Small Cell Lung Cancer[Title/Abstract])) OR (NSCLC[Title/Abstract]))) AND (((Metastasis[Title/Abstract]) OR (Metastase*[Title/Abstract])) AND (("Brain"[Mesh]) OR (((((((Brain[Title/Abstract]) OR (Encephalon[Title/Abstract])) OR (Cerebral[Title/Abstract])) OR (Intracerebral[Title/Abstract])) OR (cranial[Title/Abstract])) OR (Intracranial[Title/Abstract])) OR (Central nervous system[Title/Abstract]))))** |
| Embase | ('lung neoplasms'/exp OR 'lung neoplasms' OR (('lung'/exp OR lung) AND ('neoplasms'/exp OR neoplasms)) OR 'lung neoplasm':ti,ab,kw OR 'pulmonary neoplasm*':ti,ab,kw OR 'lung cancer*':ti,ab,kw OR 'pulmonary cancer*':ti,ab,kw OR 'cancer of the lung':ti,ab,kw OR 'cancer of lung':ti,ab,kw OR 'lung tumor*':ti,ab,kw OR 'lung carcinoma∗':ti,ab,kw) and （'brain'/exp OR brain OR encephalon:ti,ab,kw OR cerebral:ti,ab,kw OR intracerebral:ti,ab,kw OR cranial:ti,ab,kw OR intracranial:ti,ab,kw OR 'central nervous system':ti,ab,kw OR 'brain neoplasm∗':ti,ab,kw OR 'brain cancer∗':ti,ab,kw OR 'brain carcinoma∗':ti,ab,kw OR 'brain tumor∗':ti,ab,kw OR 'brain neoplasms':ti,ab,kw) and ('metastasis'/exp OR metastasis OR metastase*:ti,ab,kw) and ('radiotherapy'/exp OR radiotherapy OR radiotherap*:ti,ab,kw OR radiation:ti,ab,kw OR 'targeted radiotherap*':ti,ab,kw OR 'targeted radiation therap*':ti,ab,kw OR irradiation:ti,ab,kw OR radiosurgery:ti,ab,kw OR 'whole-brain radiotherapy':ti,ab,kw OR WBRT:ti,ab,kw) |
| Web Of Science | (Carcinoma, Non-Small-Cell Lung (topic) or Carcinoma, Non‐Small‐Cell (topic) or Carcinoma, Non Small Cell Lung(topic) or Carcinomas, Non‐Small‐Cell Lung(topic) or Lung Carcinoma*, Non‐Small‐Cell(topic) or Non Small Cell Lung Carcinoma(topic) or Carcinoma, Non‐Small Cell Lung(topic) or Non‐Small Cell Lung Cancer(topic) or NSCLC(topic)) AND ((Brain(topic) or Encephalon(topic) or Cerebral(topic) or Intracerebral(topic) or cranial(topic) or Intracranial(topic) or Central nervous system(topic) ) AND (Metastase*(topic) or Metastasis (topic) ) ) AND (Radiotherap*(topic) or Radiation(topic) or Targeted Radiotherap*(topic) or Targeted Radiation Therap*(topic) or Irradiation(topic) or Radiosurgery(topic) or whole-brain radiotherapy(topic) or WBRT(topic)) |
| The Cochrane Library | (“brain”[MeSH Terms] OR “Intracranial”:ti,ab,kw OR “Cranial”:ti,ab,kw OR “Central nervous system”:ti,ab,kw OR “Encephalon”:ti,ab,kw OR “Cerebral”:ti,ab,kw OR “Intracerebral”:ti,ab,kw OR brain neoplasm*:ti,ab,kw OR brain cancer*:ti,ab,kw OR brain tumor*:ti,ab,kw OR brain neoplasms:ti,ab,kw) AND (“Metastase* ”:ti,ab,kw OR “metastases”:ti,ab,kw) AND (“Radiotherapy”[MeSH Terms] OR “radiotherap*”:ti,ab,kw OR “radiation”:ti,ab,kw OR “radiosurgery”:ti,ab,kw OR “irradiation”:ti,ab,kw OR “targeted radiotherap”:ti,ab,kw OR “targeted radiation therap”:ti,ab,kw OR “WBRT”:ti,ab,kw OR “whole-brain radiotherapy”:ti,ab,kw ) AND (“lung cancer”[MeSH Terms] OR “lung neoplasm”:ti,ab,kw OR “pulmonary neoplasm”:ti,ab,kw OR “lung cancer*”:ti,ab,kw OR “pulmonary cancer*”:ti,ab,kw OR “cancer of lung”:ti,ab,kw OR “cancer of lung”:ti,ab,kw OR “lung tumor*”:ti,ab,kw OR “lung carcinoma*”:ti,ab,kw ) |
