# Supplementary material for: RT-based combination therapy for brain metastasis from NSCLC with non-EGFR mutation/ALK gene rearrangement: A network meta-analysis
Source: Front Oncol. 2022 Nov 28;12:1024833. doi: 10.3389/fonc.2022.1024833 (PMC9744133; doi:10.3389/fonc.2022.1024833)
Supplement: Supplementary file 2 [file DataSheet_2.doc]

**Supplementary Materials**

**eFigure 1.** Funnel plots of different endpoints.

1. HR for OS, (B) HR for iPFS, (B) HR for PFS


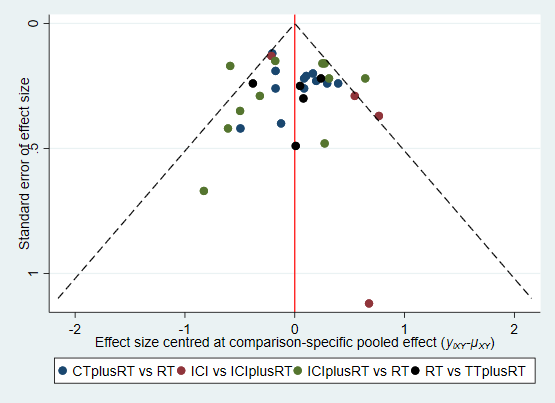

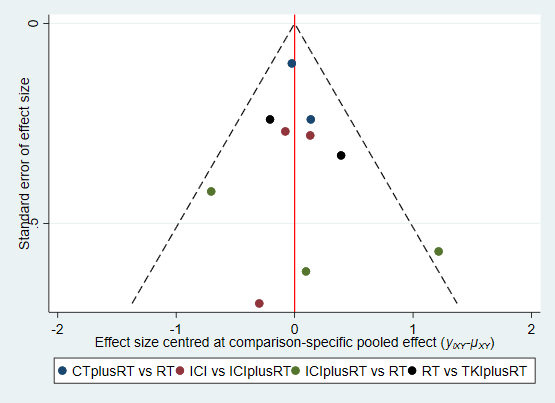

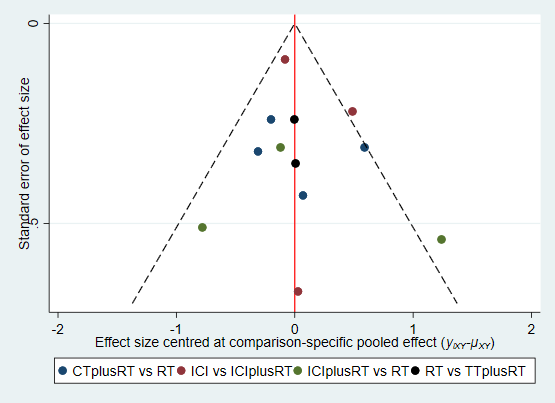


**A B C**

**eFigure 2.** Heterogeneity Analysis Charts of different endpoints.

1. OS, (B)iPFS, (C)PFS


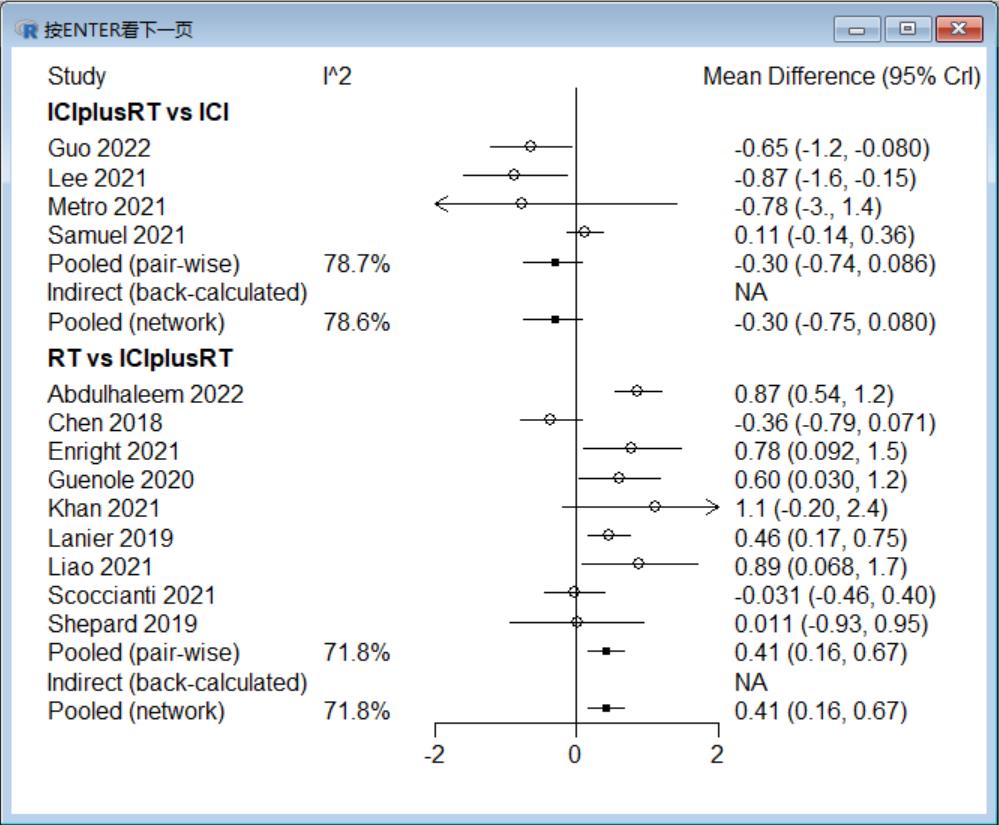

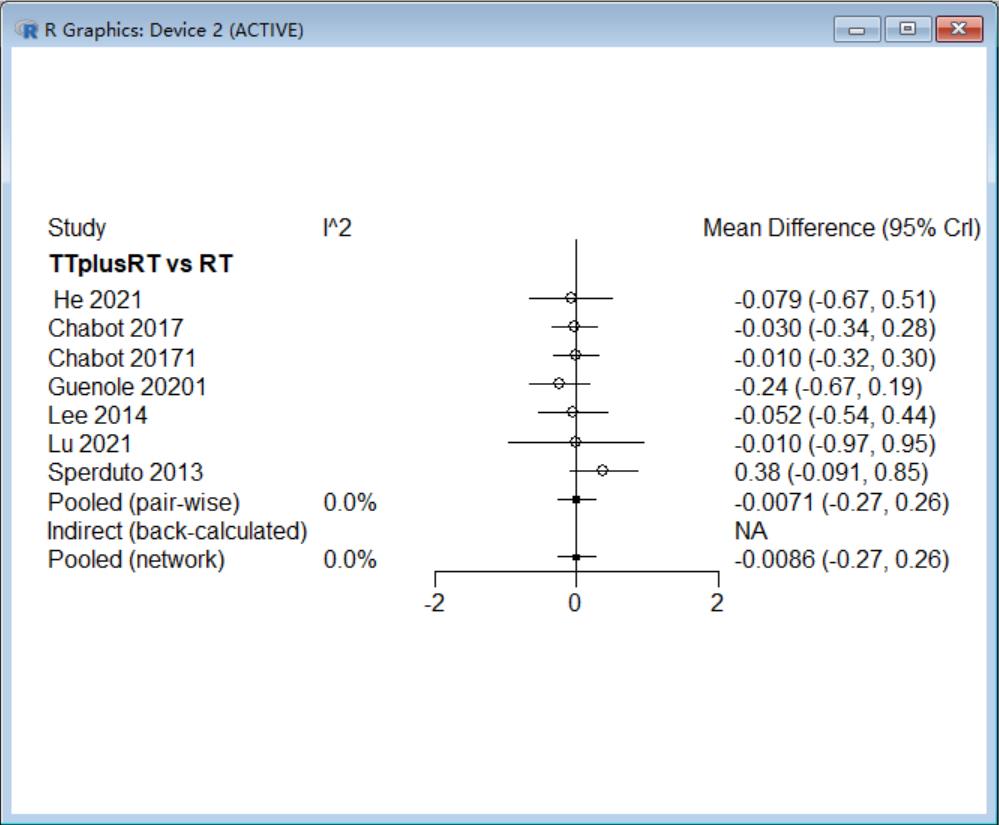

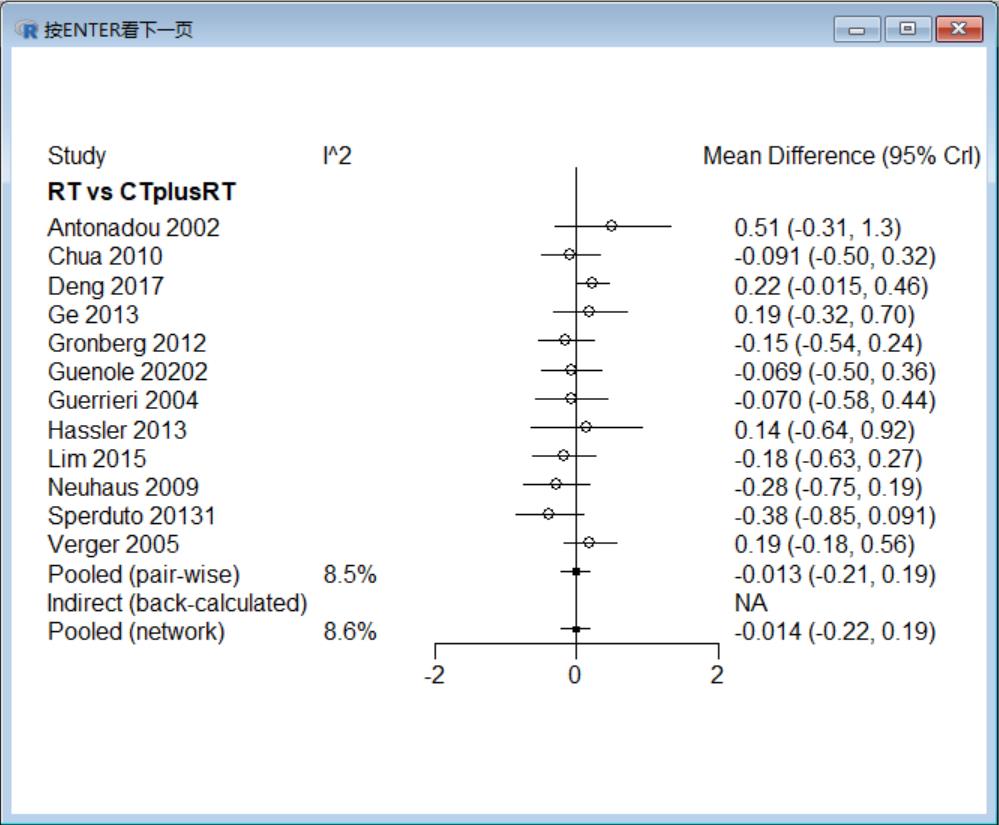


**eFigure 2A**

Continue with the picture


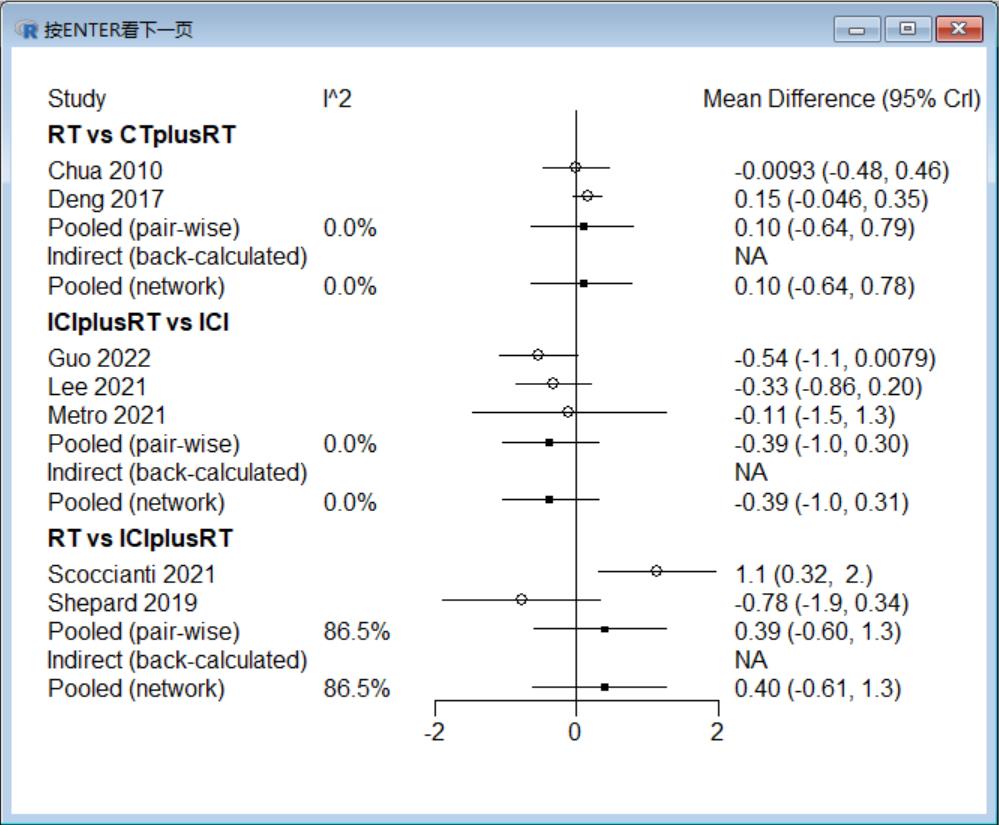

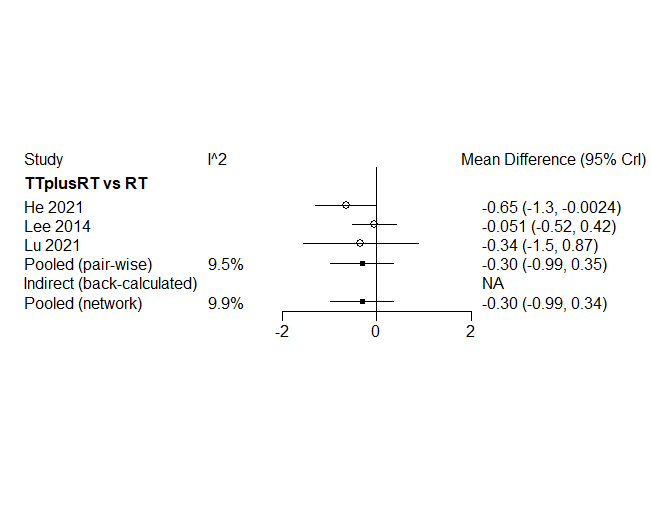


**eFigure 2B**

Continue with the picture

**
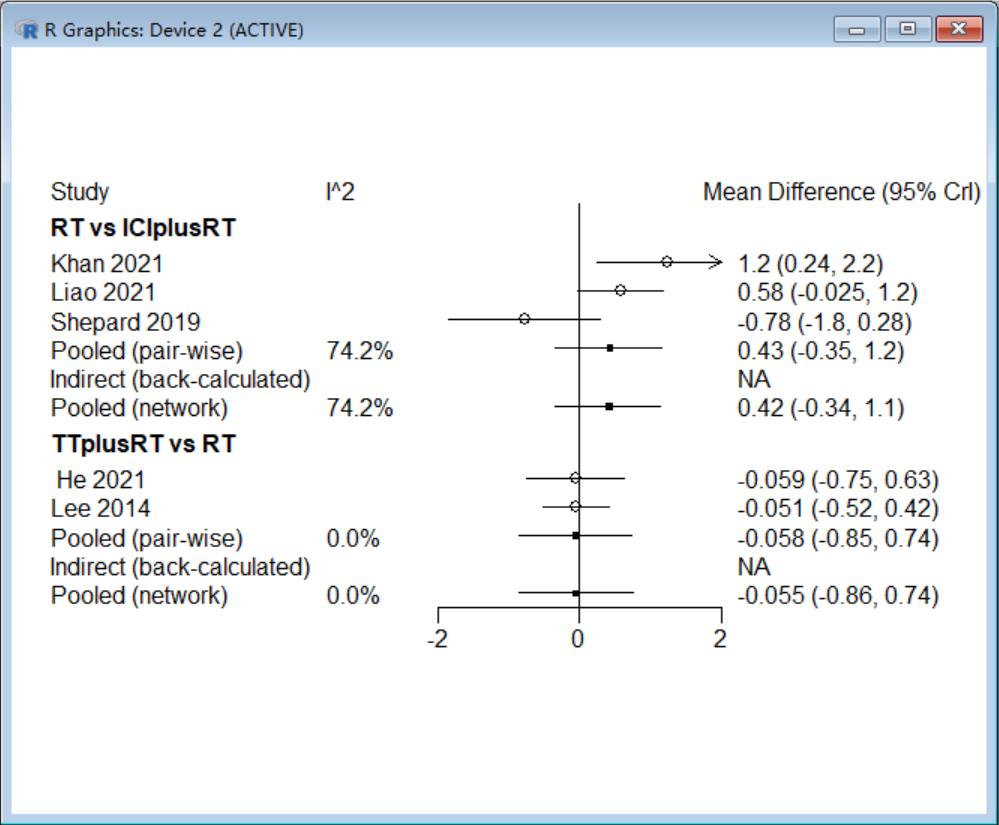

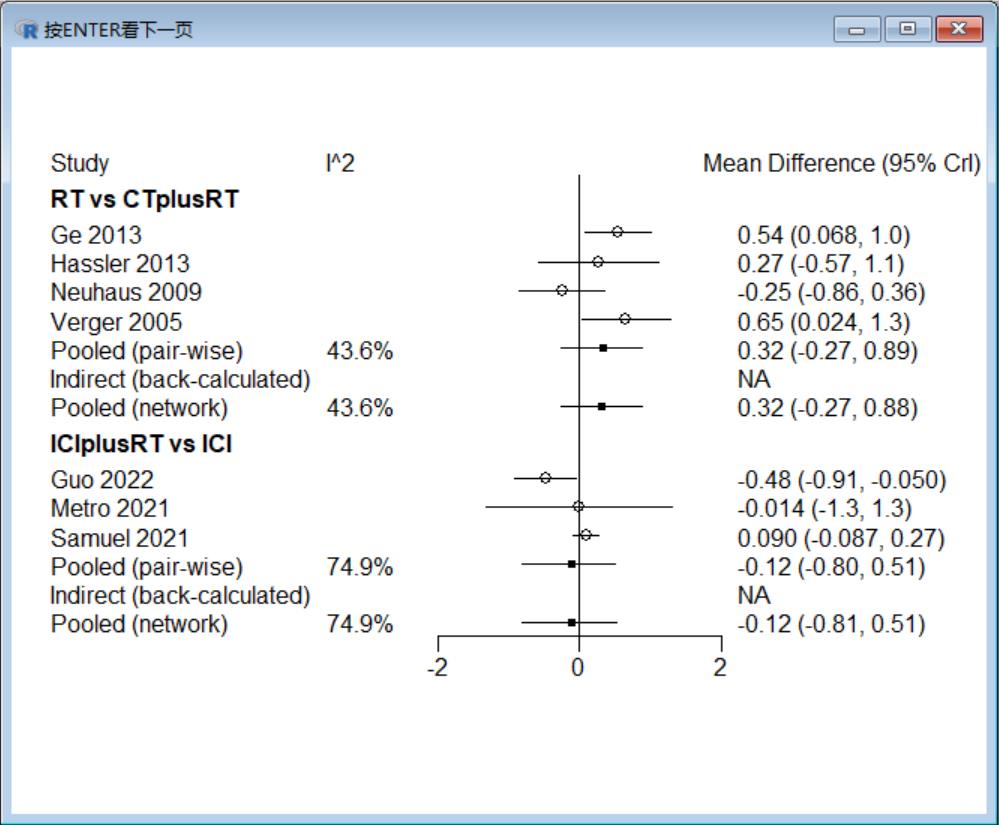
**

**eFigure 2C**

**eFigure 3.** Density charts of different endpoints.

1. OS, (B)iPFS, (C)PFS


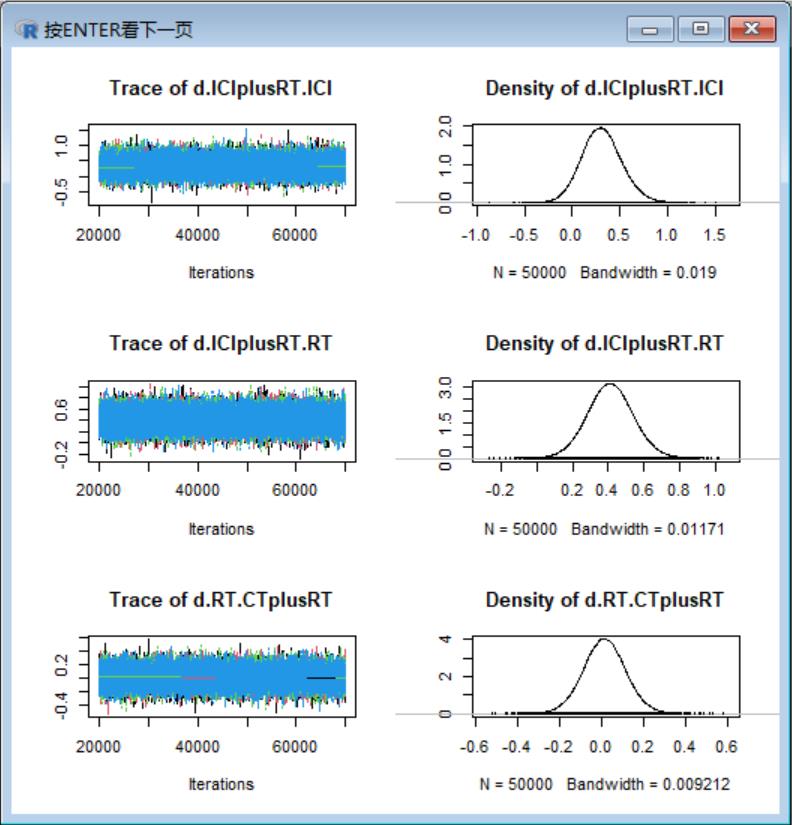

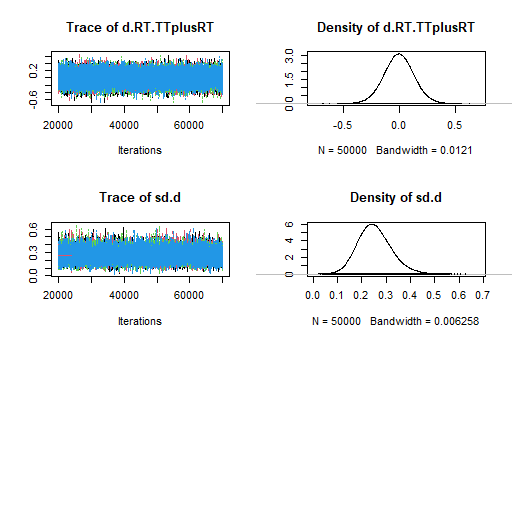


**eFigure 3A**

Continue with the picture


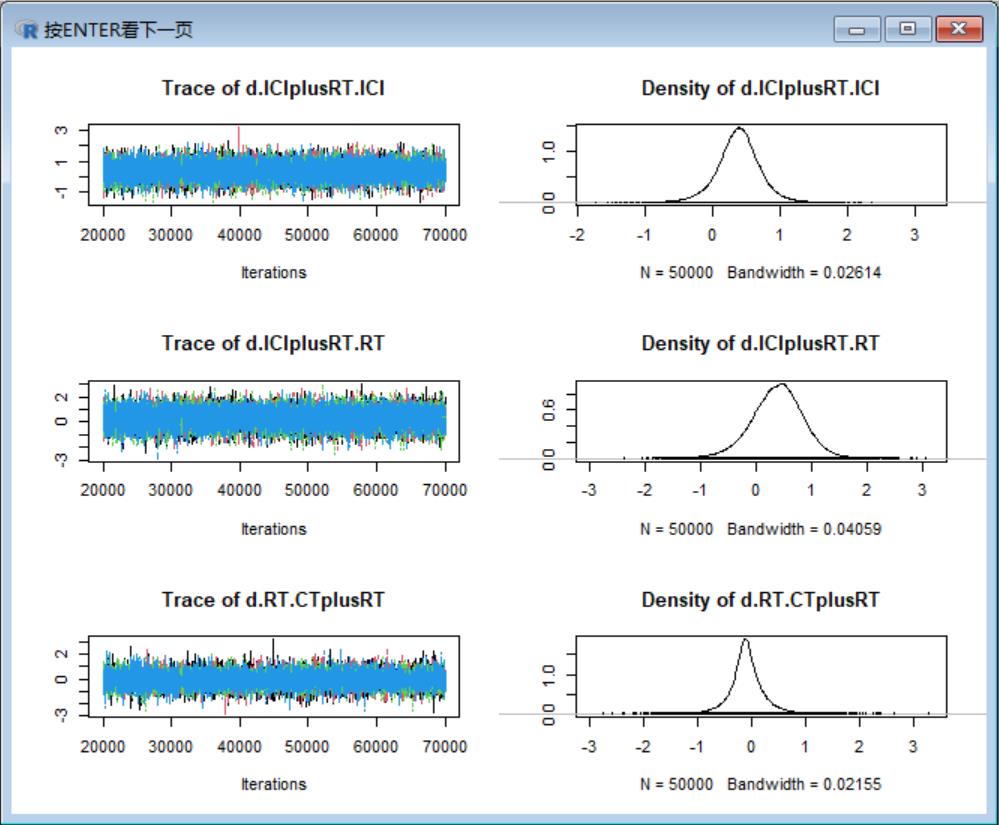

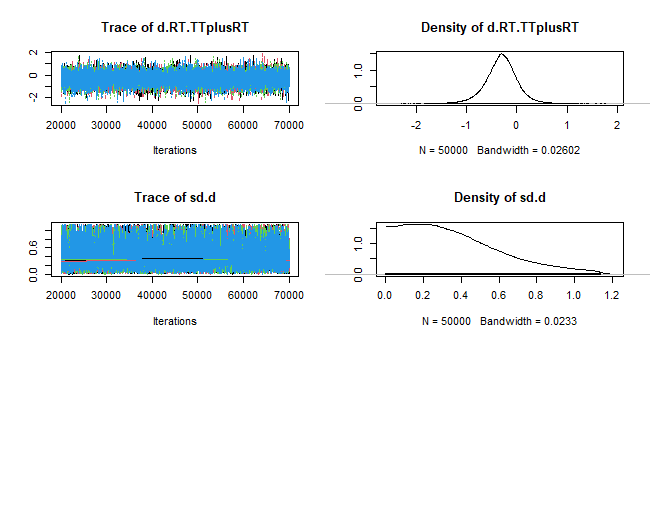


**eFigure 3B**

Continue with the picture


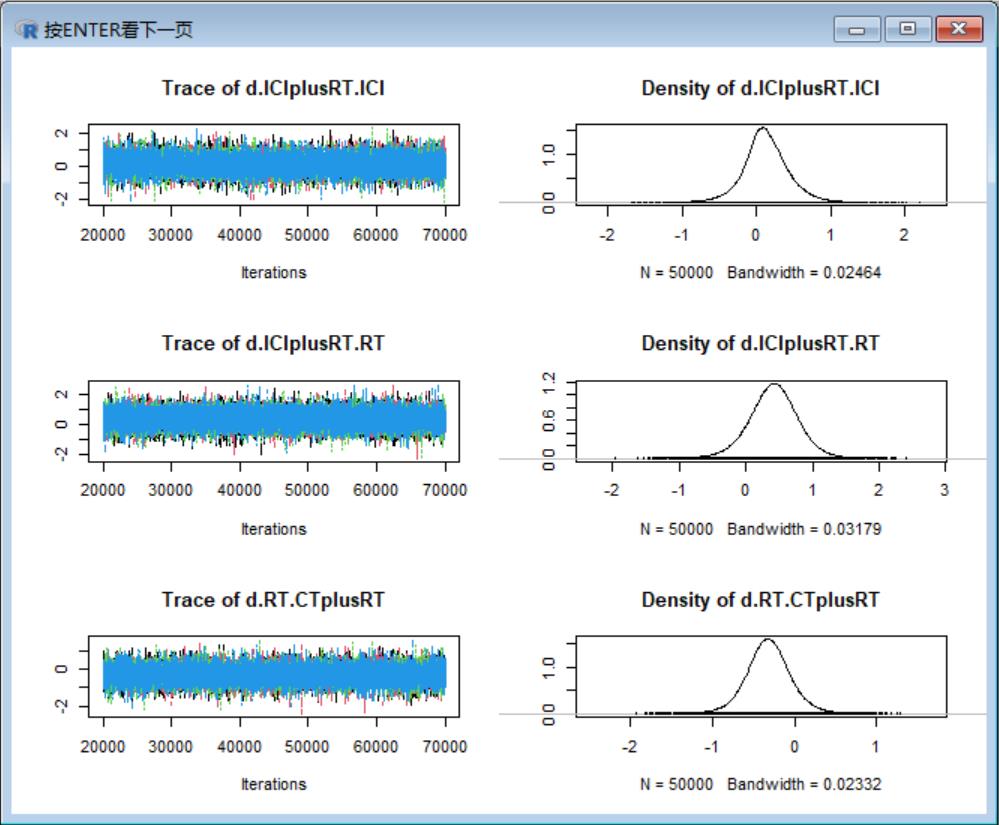

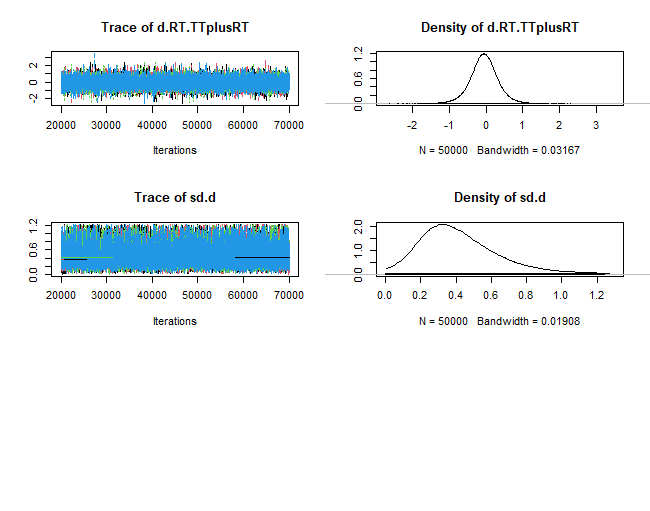


**eFigure 3C**

**eFigure4.** Convergence charts of different endpoints

1. OS, (B) iPFS, (C) PFS


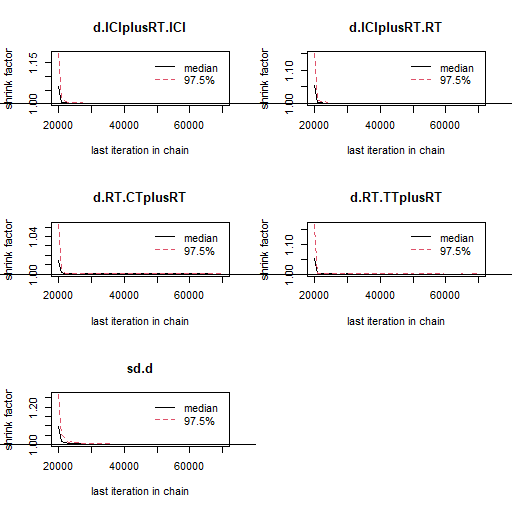

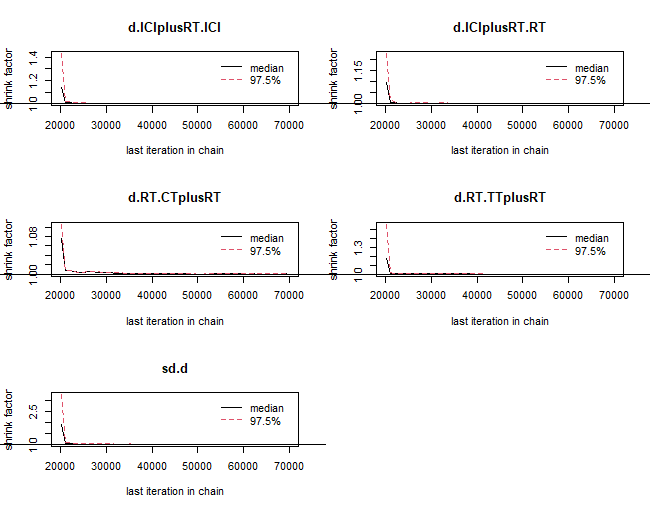

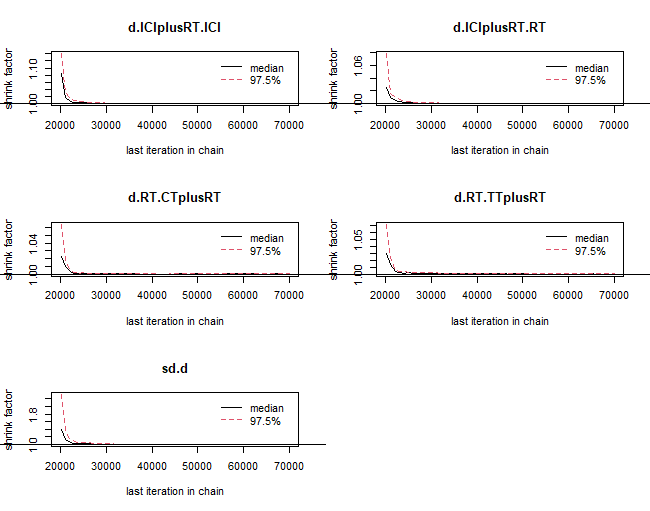


**eFigure4 A eFigure4 B eFigure4 C**
